# Supplementary figures and images for: Ligninolytic activity of the Penicillium chrysogenum and Pleurotus ostreatus fungi involved in the biotransformation of synthetic multi-walled carbon nanotubes modify its toxicity
Source: PeerJ. 2021 Mar 31;9:e11127. doi: 10.7717/peerj.11127 (PMC8019314; doi:10.7717/peerj.11127)

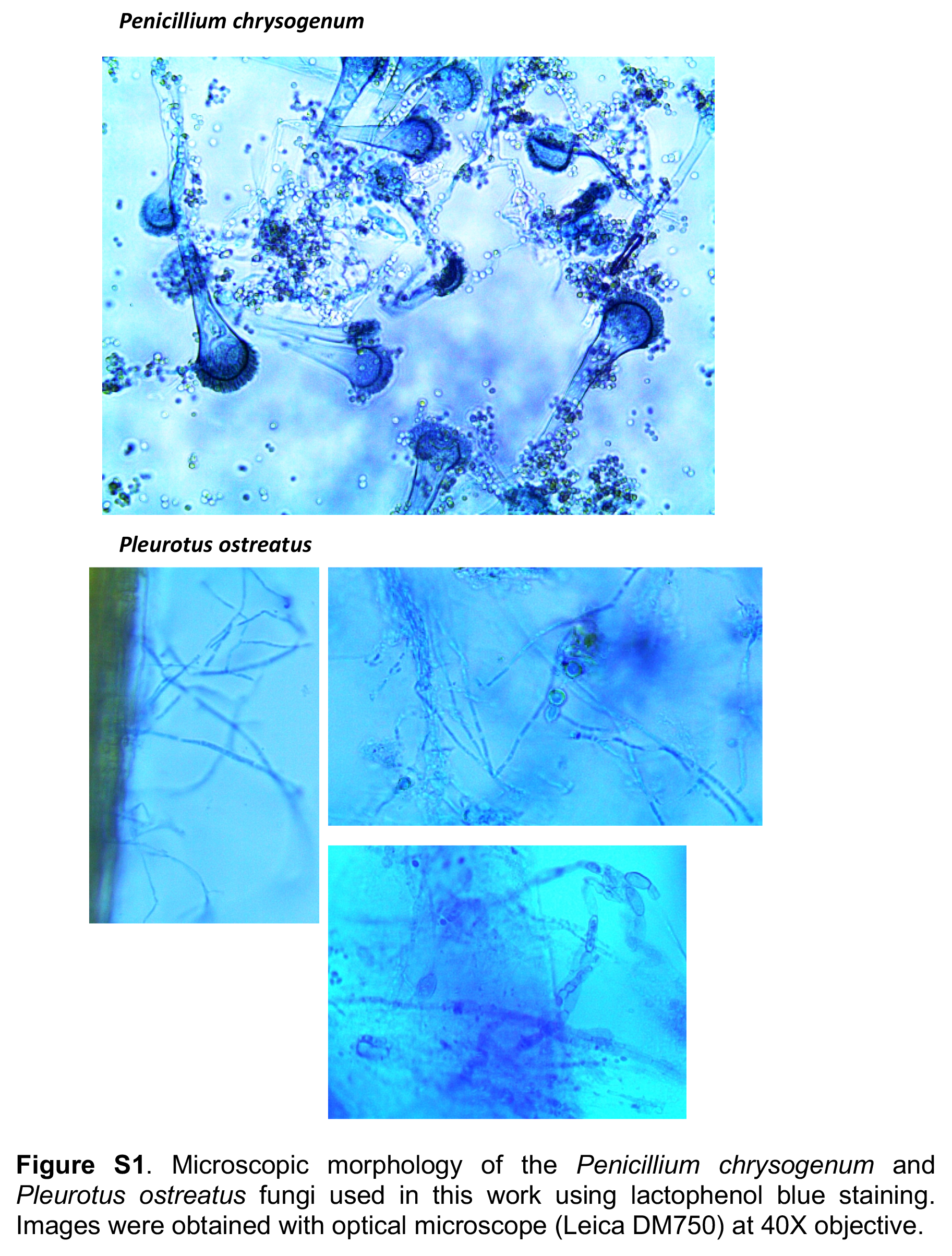

Supplement: Supplemental Information 1 — Fungi used in this work using lactophenol blue staining. Images were obtained with optical microscope (Leica DM750) at 40X objective. [file peerj-09-11127-s001.png]

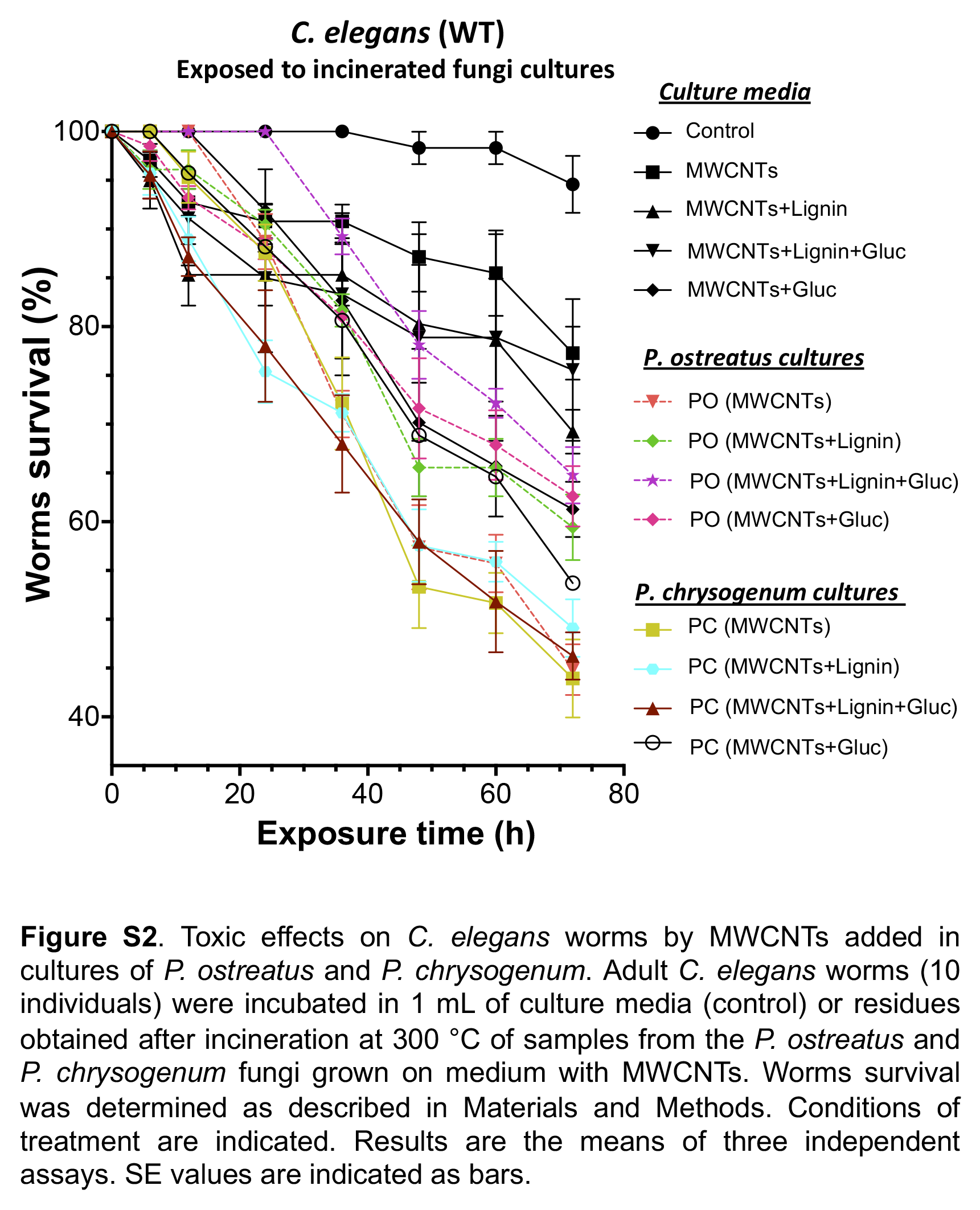

Supplement: Supplemental Information 2 — Adult C. elegans worms (10 individuals) were incubated in 1 mL of culture media (control) or residues obtained after incineration at 300 °C of samples from the P. ostreatus and P. chrysogenum fungi grown on medium with MWCNTs. Worms survival was determined as described in Materials and Methods. Conditions of treatment are indicated. Results are the means of three independent assays. SE values are indicated as bars. [file peerj-09-11127-s002.png]
